# Investigation of bacterial agents in yaws-like ulcers in children and their environment in Cameroon: An experience from the Akonolinga Health District

**DOI:** 10.1371/journal.pntd.0014579

**Published:** 2026-07-27

**Authors:** Francky Ghislain B’sop, Mama Mbouombouo, Serges Tchatchouang, Diane Kamdem Thiomo, Dell-Dylan Kenfack, Ange Patricia Kamgmo Tappe, Felix Priso Bile, Hilaire Claude Zo Evouna, Michel Kengne, Leonard Numfor, Francine Ntoumi, Jean-Paul Assam-Assam, Earnest Njih-Tabah, Veronique Penlap Beng

**Affiliations:** 1 Department of Microbiology and Medical Immunology, School Health Sciences, Catholic University of Central Africa, Yaoundé, Cameroon; 2 Higher Institute of Agriculture, Forestry, Water and Environment, University of Ebolowa, Ebolowa, Cameroon; 3 Department of Biochemistry, Faculty of Science, University of Yaoundé 1, Yaoundé, Cameroon; 4 Centre Pasteur du Cameroun, Yaoundé, Cameroon; 5 Chantal Biya International Reference center for research on the prevention and management of HIV/AIDS, Yaoundé, Cameroon; 6 Akonolinga Health District, Akonolinga, Cameroon; 7 Fondation Congolaise pour la Recherche Médicale, Brazzaville, Republic of Congo; 8 Privatdozentin, Faculty of Medicine, Institute for Tropical Medicine, University of Tubingen, Tübingen, Germany; 9 Department of Microbiology, Faculty of Science, University of Yaoundé 1, Yaoundé, Cameroon; 10 National Buruli Ulcer, Leprosy, Yaws and Leishmaniasis Control Program, Ministry of Public Health, Yaounde, Centre Region, Yaoundé, Cameroon; 11 Public Health & Epidemiology, University of Dschang, Dschang, Cameroon; Yale School of Medicine: Yale University School of Medicine, UNITED STATES OF AMERICA

## Abstract

**Background:**

Skin ulcers are health problem in children in tropical areas. Some clinically resemble yaws and this may cause inappropriate allocation of resources from the WHO yaws eradication plan. Studies have shown that bacteria other than the *Treponema pallidum* subspecies *pertenue* (bacterium causing yaws) were responsible for these lesions with certain associated risk factors. This study aimed to detect bacteria in yaws-like skin ulcers in Akonolinga Health District and the involvement of some matrices (pets, water, flies).

**Methods:**

A cross-sectional study was conducted from April to June 2023. Swabs were collected from children aged 5–15 years with yaws-like lesions and their asymptomatic contacts. Alongside this, samples were also collected from domestic animals, rivers and flies in the vicinity of participants with yaws like lesions. DNA extracted from the samples collected was used to amplify specific genes to detect the bacteria: *Treponema pallidum* subspecies *pertenue*, *Haemophilus ducreyi*, *Staphylococcus aureus* and *Arcanobacterium haemolyticum*.

**Results:**

Among the 5,931 children screened, 28 presented with yaws-like lesions. Swab samples were collected from these 28 symptomatic individuals as well as from 28 asymptomatic contacts. Among the targeted genes tested, only *Staphylococcus aureus* DNA could be amplified from the samples of symptomatic at 32.1% (9/28) and in asymptomatic at 21.42% (6/28) (p = 0.547). These bacterial DNA could not be amplified from samples from animal and environmental matrices.

**Conclusion:**

*Staphylococcus aureus* is a bacterium involved in yaws-like ulcers but also found in similar proportion among asymptomatic healthy controls. Further investigation in these skin ulcers is needed for wider diagnostic tools to manage affected population.

## Author summary

Yaws, a skin Neglected Tropical Diseases (NTDs) is targeted to be eradicated by 2030 and the primary control method involves Mass Drug Administration (MDA) with azithromycin. Without accurate diagnosis of yaws, resources can be misallocated because of its clinical similarity to other skin ulcers. Previous studies have established that a high proportion of clinically suspicious lesions often test negative for *Treponema pallidum* subspecies *pertenue*, the aetiological agent of yaws. In the Akonolinga Health District in Cameroon, this study investigated the bacterial aetiologies of yaws-like lesions as well as the possible, risk factors and contribution of environmental matrices. Approximately 6,000 children aged 5–15 years have been screened through active case searching following an integrated approach. A total of 28 individuals with yaws-like ulcers were identified with their 28 asymptomatic contacts. Swabs from lesions, intact skin and samples from animals, water, and flies were collected for PCR detection of four bacteria: *Treponema pallidum* subspecies *pertenue, Haemophilus ducreyi, Staphylococcus aureus*, and *Arcanobacterium haemolyticum*. Only *Staphylococcus aureus* was detected in 32.1% of symptomatic and 21.42% of asymptomatic children, but not in environmental and animal samples. Alongside, eight Buruli ulcer cases were identified. The findings show *Staphylococcus aureus* involvement in yaws-like ulcers and healthy controls which requires deep investigation in this Buruli ulcer-endemic region. The data suggest that the health district may not be endemic for yaws and control efforts for yaws eradication should be redirected in other areas where the disease has been laboratory-confirmed.

## Introduction

Skin conditions affect almost 900 million people each year worldwide and are the third most common reason of medical consultation [1–3]. Among them, yaws is a skin neglected tropical disease (NTD) for which 50 million cases were reported in 1952. Following integration into public health programs, the disease burden was drastically reduced and re-emerged towards the end of the 1970s [4,5]. Among the 16 countries currently endemic for yaws, the 2024 data show that 152,164 suspected yaws cases were reported from 10 countries, with 996 confirmed cases in seven countries. Cameroon is among the nine Africans countries endemic for yaws. In 2024, 1620 suspected cases were reported in Cameroon, but none were confirmed. In contrast, over the three previous years (2021–2023), a cumulative total of 123 confirmed cases were reported [6]. Ghana and Papua New Guinea alone accounted for more than 80% of the reported cases in previous reports [6]. The discrepancy between number of suspected and confirmed yaws cases highlights the gap in the diagnosis. This is problematic because most yaws cases reported to the World health Organization (WHO) are based on the clinical diagnosis. Yaws is difficult to diagnose based on clinical presentation alone, necessitating serological and molecular tools for confirmation. A substantial gap in diagnostics persists because few of these endemic countries have a diagnostic platform and most data are still clinically based [7]. Furthermore, existing laboratory data (e.g., molecular diagnostics) at the country level come from research projects, with samples often analysed outside the endemic countries. This could explain therapeutic failures that stem from challenge of distinguishing between yaws and clinically similar skin ulcers. Treatments targeted specifically for yaws may be ineffective against other similar skin ulcers [8]. As for Africa, Cameroon, Côte d’Ivoire, Congo and Ghana are endemic countries for yaws and have been part of a project to evaluate a Point-Of-Care molecular test [9,10]. This research, which is more conclusive and more decisive, shows that reported cases of yaws are not always caused by *Treponema pallidum* subspecies *pertenue*. In some cases, it is another aetiology such as *Haemophilus ducreyi* or a bacterial co-infection with *Treponema pallidum* subspecies *pertenue* in low proportion in many published studies [8,11–13].

Five years into the 2030 NTD roadmap, it is necessary to investigate areas where yaws notifications have been reported. This allows to achieve the objectives set by laboratory confirmation of cases and appropriate treatment. Previous research has identified about 15 bacterial species in the microbiome of skin ulcers similar to yaws, in which some return at varying carriage rates. In 2019 in Papua New Guinea, 15 bacteria were recorded, five of which were strongly involved in the appearance of yaws-like conditions. The micro-organisms included *Haemophilus ducreyi* with a predominance of 23%, *Treponema pallidum* subspecies *pertenue* for a rate of 16%, *Streptococcus dysgalactiae (*12%), *Arcanobacterium haemolyticum (8%)* and *Corynebacterium diphteriae* (8%). Theshotgun metagenomics were used and these results suggest a varied aetiology for skin ulcers in yaws endemic areas [14]*.* In 2024, in Ghana and the Solomon Islands *Staphylococcus spp.* and *Streptococcus spp*. were detected in skin conditions that could be confused with yaws or *Haemophilus ducreyi* ulcers. These data could not be interpreted as a definitive causation. The study show that 15 bacteria are also recorded in the microbiome of these ulcers. They include *Campylobacter ureolyticus* with 42% detection, *Streptococcus pyogenes* at 36%, *Arcanobacterium haemolyticum* at 34%, and *Corynebacterium diphtheriae* present at 21% [15]. In 2021 and 2023 in Cameroon, two studies highlighted the predominance of *Haemophilus ducreyi* in the appearance of skin lesions similar to yaws. *Haemophilus ducreyi* was identified at 49.6% compared to 23.4% for *Treponema pallidum* subspecies *pertenue* in the first study [11]. In 2023 in 14 districts of Cameroon, 30.3% of *Haemophilus ducreyi* and 5.2% of *Treponema pallidum* subspecies *pertenue* [12].

In addition, environmental factors such as water, insects, and fomites have been identified as reservoirs and matrices of the bacteria responsible for yaws-like skin ulcers. In 2017 in Papua New Guinea, 90% of flies were carriers of *Haemophilus ducreyi* and half of these same flies carried *Treponema pallidum* subspecies *pertenue*. Similarly, out of six bed sheets were collected by swabbing 10x10 cm across five separate areas during this research in Papua New Guinea. Two sheets were found to carry *Haemophilus ducreyi* against a bed sheet containing *Treponema pallidum* subspecies *pertenue* [16]. In Cameroon, 8.6% of asymptomatic participants carrying the *Haemophilus ducreyi* were recorded in 2023 (10)*. Haemophilus ducreyi* was detected on both clothing (13.3%) and in flies (27%) while *Treponema pallidum* (detection at species level) was tested positive in flies (2.6%) [17]. Fly-to-human mechanical transmission of these bacteria has not been definitively proven. While the bacteria have been detected in flies, the precise epidemiological importance of flies or fomites in transmission remains unknown.

The Akonolinga health district in Cameroon is known for its endemicity to Buruli ulcer [18,19]. Notification data shows 21 cases of yaws recorded in March 2023 (with a total of 69 suspected cases during the year 2023) based on clinical diagnosis (data from the Akonolinga health district). Exploring yaws-like idiopathic ulcers is crucial. This research aimed to strengthen the hypothesis of a bacterial co-detection or even a bacterial involvement other than *Treponema pallidum* subspecies *pertenue* in the appearance of yaws-like ulcers in the Akonolinga health district, as well as the potential sources of bacterial spread through animals and environment.

## Methods

### Ethical consideration

Prior to the start of the field study, the necessary authorizations were obtained, including those of the study site (Akonolinga health district and the Ministry of Basic Education) and the ethical approval of the Centre Regional Human Health Research Ethics Committee (CRERSH/Centre, N°0016/CRERSHC/202). Written informed consent was obtained from the legal representatives of all participants. In addition, children aged 11–15 years gave their assent consisting of a verbal agreement and a fingerprint prior to enrolment.)

### Type of study, population, period, inclusion criteria

From April to June 2023, an active search for skin ulcers clinically similar to yaws was conducted among children aged 5–15 years in primary schools and communities within the Akonolinga health district, using an integrated approach. The integrated approach implemented involved simultaneous collection of data on yaws-like ulcers and referral of other reported skin NTDs to the health district. Participants included children presenting with one or more circular ulcers of at least 1 cm in diameter, as well as children without yaws like lesion (asymptomatic) in the vicinity of individual presenting with this type of ulcer. In this stud***y***, a contact was defined as any child within the same age group and sex who shared a common environment with the case, specifically including classmates, household members, neighbours or playmates. In addition, waters, flies and pet animals were sampled for this research.

### Study site

The Akonolinga health district is located in the Nyong and Mfoumou division within the central region of Cameroon. The health district comprises 13 health areas, covering a surface area of approximately 4,300 km^2^ with an estimated population of 90,625 in 2022 (health district data, 2022). It encompasses three administrative entities: Akonolinga, Endom, and Mengang sub-divisions. The inhabitants of the Akonolinga rely primary on fishing, hunting and agriculture for their livelihoods. The health district is one of the main endemic areas of Buruli ulcer in Cameroon. This health district is among the 152 districts reporting suspected cases of yaws in Cameroon. However, this status of suspected endemicity remains unconfirmed [7]. Data from District Health Information Software 2 (DHIS 2) show that 10, 13, and 67 suspected cases of yaws were reported in 2020, 2021 and 2022 respectively.

### Sample collection

Swab samples were collected from children aged 5–15 years presenting with skin lesion clinically similar to yaws, as well as from the intact skin of their asymptomatic contact. For yaws-like lesions, sample collection was performed by firmly pressing the head of the Dacron swab into the center of the lesion and rotating it to absorb the serous fluid**.** In case a symptomatic participant had multiples contacts, the contact with whom the patient reported the closest social proximity or highest frequency of interaction was selected. All contacts selected were sampled on the same anatomical site as the patients consisting of swabbing an area of 2x2 cm of their intact skin using Dacron swab moistened with sterile saline. In the vicinity of human participant with skin lesion, swabs were collected from available domestic animals at the perianal region, any active wound, and the oral cavity. The dogs enrolled included those found near human symptomatic participants. The canine belonged either to the participant’s household or to the neighbourhood. Whole flies were captured and trapped around the patient with the yaws-like lesion using a twig broom. A pool of four flies collected in 1 mL of lysis buffer (10mM Tris pH 8.0, 0.1M EDTA pH 8.0, 0.5% sodium dodecyl sulfate) in the same environment was considered as a fly sample.

For water samples (rivers), a volume of 15 mL was collected in a falcon tube from each water source closest to the symptomatic participant. Water sources were selected based on community usage for drinking, washing, bathing and swimming purposes.

The swab samples obtained were stored in 1.5 mL screw-top microtubes containing 500 µL of lysis buffer (10mM Tris pH 8.0, 0.1M EDTA pH 8.0, 0.5% SDS) for DNA stabilization prior to shipment for molecular testing**.**

The samples were transported in a cool box from the field to the Akonolinga district hospital, where they were stored at 4°C for 3–4 days. They were subsequently transferred to the Biotechnology Centre of, University of Yaounde 1 (Nkolbisson) in a cool box equipped with a refrigeration unit, and stored at -20°C until testing.

### Nucleic acid amplification

#### Sample processing.

All samples were removed from the freezer and allowed to thaw at room temperature. For the swabs collected, each 1.5 mL microtube was vortexed, and 200 µL of suspension was taken for DNA extraction. The water samples were centrifuged at 4000 rpm for 10 minutes. The supernatant was then decanted, and the resulting pellet was resuspended in

500 µL of lysis buffer (10mM Tris pH 8.0, 0.1M EDTA pH 8.0, 0.5% SDS) prior to DNA extraction. The pooled flies were ground using a mortar and a pestle, after which 200 µL of the vortexed homogenate was used for DNA extraction.

#### DNA extraction.

DNA was extracted using the Qiagen QIAamp DNA Mini Kit (Qiagen, Hilden, Germany) according to the manufacturer’s protocol. Briefly, 200 μL of lysed sample after vortexing were processed by adding 20 μL of proteinase K and incubating the mixture at 56°C for 1 hour. Following incubation, 200 μL of AL buffer were added and another incubation at 70°C for 10 minutes was done. A 200 μL of absolute ethanol was added and a centrifugation followed. The mixture was then transferred to spin columns and washed sequentially with 500 μL of AW1 and 500 μL of AW2. DNA was eluted by adding 100 μL of molecular-grade water to the spin column, and the resulting DNA was stored at -20°C. The procedure was the same for all collected samples.

#### Polymerase chain reactions.

For *Treponema pallidum* subspecies *pertenue*, *Staphylococcus aureus* and *Arcanobacterium haemolyticum*, DNA amplification was performed by end-point PCR targeting the *Tp0548, nuc* and *aln* genes, respectively (see Table 1 for more details). Then, the amplified products were detected on agarose gel electrophoresis. When *Tp0548* gene was detected, a second PCR for subspecies *pertenue* was required by targeting *TprL* gene*.* Prior to this step, sample quality, presence of host DNA, and absence of amplification inhibitors in the extracted DNA were assessed by amplifying a 268 bp fragment of the ß2-microglobulin (beta-2-M) gene, a housekeeping gene using the primers beta-2-M_F (5’-CAACTTCATCCACGTTCACC-3’) and

**Table 1:** Polymerase chain reactions conditions.

Pathogen	Target gene	Master mix volume	Oligonucleotides	DNA volume	Amplification program	Amplicon size (bp)	platform	Reference
*Staphylococcus aureus*	*Nuc*	45 µL	nuc-F: 5’-GCGATTGATGGTGATACGGTT-3’ nuc-R: 5’-AGCCAAGCCTTGACGAACTAAAGC-3’	5 µL	2 min 95°C, 37x (1min 94°C, 30 sec 55°C, 1 min 30 sec 72°C) 3 min 30 sec 72°C, tenir à 4°C	270 bp	Applied Biosystem gene AMP	[21]
*Treponema pallidum* subspecies *pertenue*	*Tp0548*	45 µL	Tp0548 S: 5’-GGTCCCTATGATATCGTGTTCG-3’ Tp0548 R: 5’-CGTTTCGGTGTGTGAGTCAT-3’	5 µL	2 min 95°C, 37x (1min 95°C, 1min 15 sec 55°C, 1 min 72°C) 10 min 72°C, tenir à 4°C	131 bp	Applied Biosystem gene AMP	[22]
*Arcanobacterium haemolyticum*	*Aln*	45 µL	aln-F: 5’-GTCAAGTTATGCCGGGAATG-3’ aln-R: 5’-CGATGTTCTTGAACCAAGG-3’	5 µL	2 min 94°C, 30x (5min 94°C, 1 min 55°C, 1 min 72°C) 5 min 72°C, tenir à 4°C	1984 bp	Applied Biosystem gene AMP	[23]
*Haemophilus ducreyi*	*Hhda*	20 µL	hhdA-F: 5’-AATCGTTAACTGCGGGATTAGG-3’ hhdA-R: 5’-CAATAGACACATTATCGCCCTTTAAA-3’ hhdA-P: 5’-ATGGCCATGGTAGTGAGGTAAATCAG GCTGT-3’	5 µL	3 min 95°C, 35x (5sec 95°C, 25 sec 60°C,)	93 bp	Applied Biosystems Kit Quantstudio 7	[24]

beta-2-M_R (5’-GAAGAGCCAAGGACAGGTA-3’) [20]. *Haemophilus ducreyi* was detected by real-time PCR by amplifying the *hhda* gene, which codes for haemolysin.

For each target, in-house positive controls consisting of known DNA extracts were used. The negative controls consisted of negative DNA extraction control (molecular grade water processed alongside the samples to monitor the extraction process) and a no-template control (molecular grade water).

The different amplification programs for each target are recorded in the Table 1:

#### Data analysis.

All data were entered into Excel, and simple descriptive statistics were used to summarize sample and patient characteristics, as well as analytical results by amplification. The Statistical Package for Social Sciences software (version 22.0, SPSS Inc., Chicago, IL, USA) was used for the data analysis. All categorical variables were compared using the Fisher’s exact test. A p-value ˂ 0.05 was considered significant.

## Results

### Study population, yaws-like ulcer prevalence and detection of bacteria in specimens

We screened 5,931 children, including 3,215 boys (54.21%) and 2,716 girls (45.79%) from 98 villages and 21 schools. From them, we obtained 28 participants with ulcers similar to yaws, with a median age of 9 years, and 28 contacts aged between 5 and 15 years (Table 2). We collected potential transmission agents including 6 water samples, 5 dog samples and 4 pool of flies (more details in the S1 Table). The prevalence of skin ulcers clinically similar to yaws was 0.5%. During active case searching, eight suspected cases of Buruli ulcers were identified and were reported to the health district for the management. Additional information on reported Buruli ulcers is available in the S2 Table.

**Table 2:** Characteristics of population screened and bacteria detected in yaws-like ulcers in Akonolinga health district in Cameroon.

Sub-division	Health area	People screened in schools (in communities)	Number of yaws-like ulcers in schools (communities)	Number of yaws-like ulcers in males (females)	Location of the ulcer	Detection of *S. aureus* in yaws-like ulcers: male (female); [% of positive PCR males: females]	Detection of *S. aureus* in contacts: male (female); [% of positive PCR males:females]
Akonolinga	Yeme-Yeme	391 (490)	17 (3)	14 (6)	Lower limb	5 (0); [25%: 0]	4 (1); [20%: 5%]
	Akonolinga urbain	520 (611)	0 (3)	2 (1)	Lower limb	2(0); [66.67%: 0]	0 (0)
					Upper limb	0(0)	
	Djoudjoua	190 (230)	1(0)	1(0)	Lower limb	0(0)	0(1); [0: 100%]
	Emvane-sso	0 (1241)	0 (3)	3 (0)	Lower limb	1 (0); [33.33%: 0]	0 (0)
	Abem	200 (318)	0 (0)	0 (0)	/	0 (0)	0 (0)
	Zalom	97 (0)	0 (0)	0 (0)	/	0 (0)	0 (0)
	Ekoudou	0 (199)	0 (0)	0 (0)	/	0 (0)	0 (0)
	Mengueme-ssi	0 (345)	0 (0)	0 (0)	/	0 (0)	0 (0)
Mengang	Mengang	271(0)	1(0)	1(0)	Trunk	1(0); [100%: 0]	0(0)
	Ebolakounou	100 (0)	0 (0)	0 (0)	/	0 (0)	0 (0)
Endom	Endom	0 (63)	0 (0)	0 (0)	/	0 (0)	0 (0)
	Edjom	0 (106)	0 (0)	0 (0)	/	0 (0)	0 (0)
	Akak	0 (559)	0 (0)	0 (0)	/	0 (0)	0 (0)
Total		1769(4162)	19(9)	21 (7)	/	9(0); [32.14%: 0]	4(2); [14.29%: 7.14%]

*Haemophilus ducreyi, Arcanobacterium haemolyticum* and *Treponema pallidum* subspecies *pertenue* were not detected in any human samples (symptomatic or asymptomatic), nor were they found in environmental matrices, flies, or domestic animals.

*Staphylococcus aureus* was detected in 9 participants with yaws-like ulcers out of the 28 sampled (32.14%) and in 6 asymptomatic individuals (21.43%) with p = 0.547. Detection of *Staphylococcus aureus* was significantly higher in males (42.85%) compared to females (9.52%), p = 0.031. At least one case of skin ulcer associated with *Staphylococcus aureus* was recorded in 4 of the 13 health areas screened with varying proportions: Mengang, Akonolinga urbain, Emvane-sso and Yeme-Yeme (see Table 2). Colonization of *Staphylococcus aureus* in asymptomatic people has been identified in three health areas: Mengang, Djoudjoua and Yeme-Yeme.

## Discussion

In the 2030 WHO roadmap for yaws eradication, a crucial aspect is the laboratory confirmation of suspected cases [25,26]. This investigation was carried out in the Akonolinga health district, known for its endemicity to Buruli ulcer, in order to determine whether yaws is co-endemic, and alternatively to look for other pathogens as aetiological agents of skin ulcers clinically similar to yaws.

We found a 0.5% prevalence of skin ulcers similar to yaws in the health district which is low compared to other yaws endemic health districts in Cameroon such as Djoum, Mbang, Abong-Mbang, Sangmelima, and Yokadouma with prevalence over 1% [10]. None of the 28 suspected cases of yaws were PCR-confirmed. This finding aligns with previous studies conducted across nine health districts out of 12 where yaws has been reported endemic but tested negative [10].

With the exception of *Staphylococcus aureus*, with similar detection rate in symptomatic participants compared to asymptomatic individuals, the other bacteria were not detected in the samples collected. These results are similar to the findings in 2024 and 2019 in yaws-endemic countries [14,15], where approximately 26% and 81% of samples were tested positive to *Staphylococcus aureus* in Papua New Guinea, Ghana and the Solomon Islands respectively. Those baseline studies performed broad molecular sequencing, whereas the current study utilized targeted PCR. *Staphylococcus aureus* is a common pathogen in chronic wounds, with various detection rates across diverse geographical regions; ranging from 30% to 93% [27–29]. The statistics show that *Staphylococcus aureus* was more detected in boys than girls, suggesting that boys have higher colonisation rates than females. There was no significant difference between symptomatic and asymptomatic participants. In agreement with the aforementioned research, and given the absence of a significant difference between symptomatic and asymptomatic participants, presence of *Staphylococcus aureus* warrants in-depth investigation into its pathogenic role, carriage and prevalence in yaws-like lesions in Cameroon.

In addition, some bacteria detected in these tropical lesions such as *Arcanobacterium haemolyticum*, *Haemophilus ducreyi* and *Treponema pallidum* subspecies *pertenue* [9,14,15] have not been identified in this part of central Cameroon. None of the targeted bacteria were detected in the matrices studied (flies, waters, dogs). Given the exceptionally small animal and environmental sample sizes, it remains a challenge to determine whether these matrices serve as constant reservoirs of the agents responsible for tropical skin ulcers. However, the studies conducted on the Lihir Islands detected *Haemophilus ducreyi* and *Treponema pallidum* subspecies *pertenue* in human and environmental samples [16]. In Cameroon, DNA of these bacteria have been evidenced in flies and fomites [17]*.*

The main limitation of this work could be the small volume of water analysed for environmental pathogen detection compared to similar studies that used larger sample volumes [30,31]. The relatively small size of the clinical lesions within the screened population may be another limitation that affects statistical analyses. However, the findings can be also interpreted as impact of previous public health efforts to fight against diseases in the health district.

Our results demonstrates that *Treponema pallidum* subspecies *pertenue* and *Haemophilus ducreyi* are not the sole aetiological agents of skin lesions clinically similar to yaws. These findings highlight the need to to a re-evaluate suspected cases and surveillance of similar emerging tropical ulcers. Additionally, the integrated approach facilitated the detection and management of Buruli ulcer cases in the health district, thereby strengthening the NTD surveillance system.

## Conclusion

This work shows that only *Staphylococcus aureus* was detected in similar proportions among both symptomatic participants and healthy controls in the Akonolinga health district. It actually suggests that no definitive microbial causal agent has been identified. Skin ulcers clinically resembling yaws in this health district are driven by aetiologies other than *Treponema pallidum* subspecies *pertenue*, highlighting the urgent need for wider diagnostic tools to prevent the misallocation of national NTD resources.

## Supporting information

S1 Table Akonolinga. Legend: PCR DNA quality = Polymerase Chain Reaction targeting beta microglobulin used as an internal control, its detection during amplification not only confirms the human origin of the sample, DNA extraction but also reflects the absence of an inhibitor; PCR *S aureus* = Polymerase Chain Reaction targeting *Staphylococcus aureus*; PCR yaws = Polymerase Chain Reaction targeting *Treponema pallidum* for yaws diagnosis; PCR H ducreyi = Polymerase Chain Reaction targeting *Haemophilus ducreyi*; PCR A haemolyticum = Polymerase Chain Reaction targeting *Arcanobacterium haemolyticum.*
(XLSX)

S2 Table BU. Legend: BU = Buruli ulcer.
(XLSX)
